# Does Vitamin C Influence Neurodegenerative Diseases and Psychiatric Disorders?

**DOI:** 10.3390/nu9070659

**Published:** 2017-06-27

**Authors:** Joanna Kocot, Dorota Luchowska-Kocot, Małgorzata Kiełczykowska, Irena Musik, Jacek Kurzepa

**Affiliations:** Chair and Department of Medical Chemistry, Medical University of Lublin, 4A Chodźki Street, 20-093 Lublin, Poland; dorota.luchowska-kocot@umlub.pl (D.L.-K.); malgorzata.kielczykowska@umlub.pl (M.K.); irena.musik@umlub.pl (I.M.); jacek.kurzepa@umlub.pl (J.K.)

**Keywords:** vitamin C, Alzheimer’s disease, Parkinson’s disease, Huntington’s disease, multiple sclerosis, amyotrophic sclerosis, depression, anxiety, schizophrenia

## Abstract

Vitamin C (Vit C) is considered to be a vital antioxidant molecule in the brain. Intracellular Vit C helps maintain integrity and function of several processes in the central nervous system (CNS), including neuronal maturation and differentiation, myelin formation, synthesis of catecholamine, modulation of neurotransmission and antioxidant protection. The importance of Vit C for CNS function has been proven by the fact that targeted deletion of the sodium-vitamin C co-transporter in mice results in widespread cerebral hemorrhage and death on post-natal day one. Since neurological diseases are characterized by increased free radical generation and the highest concentrations of Vit C in the body are found in the brain and neuroendocrine tissues, it is suggested that Vit C may change the course of neurological diseases and display potential therapeutic roles. The aim of this review is to update the current state of knowledge of the role of vitamin C on neurodegenerative diseases including Alzheimer’s disease, Parkinson’s disease, Huntington’s disease, multiple sclerosis and amyotrophic sclerosis, as well as psychiatric disorders including depression, anxiety and schizophrenia. The particular attention is attributed to understanding of the mechanisms underlying possible therapeutic properties of ascorbic acid in the presented disorders.

## 1. Introduction

**Figure 1:**
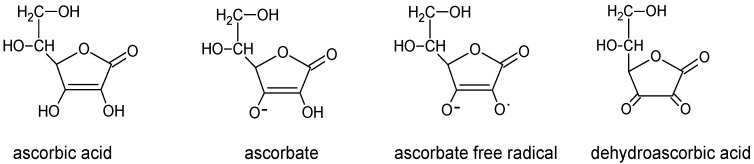
Forms of vitamin C occurring in organisms.

Vitamin C (Vit C, ascorbic acid) belongs to a group of water-soluble vitamins. In organisms, Vit C can exist in two forms: reduced—the exact ascorbic acid (AA) which in physiological pH occurs in its anion form of an ascorbate—and oxidized one—dehydroascorbic acid (DHA), which is a product of two-electron oxidation of AA (Figure 1). In the course of metabolic processes an ascorbate free radical can be produced as a result of one-electron oxidation. This variety may subsequently undergo dismutation forming ascorbate and DHA [1].

Mammalian organisms are generally capable of synthesizing Vit C themselves. However, some species like fruit bats, guinea pigs, other primates and humans are deprived of this ability due to the lack of l-gulono-1,4-lactone oxidase enzyme which is an element of the metabolic pathway responsible for synthesis of ascorbic acid from glucose [1,2]. Moreover, Vit C is not produced by intestinal microflora [3]. The above facts make these organisms strictly dependent on dietary intake. The recommended Vit C daily intake was established as 60 mg with the reservation that in smokers this value should be increased up to 140 mg [4]. According to the later recommendations, Vit C consumption should be 75 (women) and 90 (men) mg per day, whereas in smokers this value ought to be increased by 35 mg per day [3,5,6]. 

Vit C is a nutrient of greatest importance for proper functioning of nervous system and its main role in the brain is its participation in the antioxidant defense. Apart from this role, it is involved in numerous non-oxidant processes like biosynthesis of collagen, carnitine, tyrosine and peptide hormones as well as of myelin. It plays the crucial role in neurotransmission and neuronal maturation and functions [7]. For instance, its ability to alleviate seizure severity as well as reduction of seizure-induced damage have been proved [8,9]. On the other hand, disruption of vitamin C transport has been shown to contribute to brain damage in premature infants [10]. Furthermore, Vit C treatment has been reported to ameliorate neuropathological alterations as well as memory impairments and the neurodegenerative changes in rats exposed to neurotoxic substances like aluminum or colchicine [11,12].

Consequently, the growing interest in the issue of vitamin C deficiency, as well as vitamin C treatment in the nervous system diseases, was observed for many years. These facts made us decide to update the current state of knowledge of the role of Vit C in neurodegenerative diseases including Alzheimer’s disease, Parkinson’s disease, Huntington’s disease, multiple sclerosis as well as amyotrophic sclerosis, as well as in psychiatric disorders including depression, anxiety disorders and schizophrenia. 

## 2. Methods

To review the literature on brain Vit C transport/distribution and its function in central nervous system, PubMed and Scopus databases were searched using the following search terms: (vitamin C OR ascorbic acid) AND (central nervous system OR CNS) or (vitamin OR ascorbic acid) AND brain, separately. 

To review the literature on the role of Vit C in neurodegenerative diseases and psychiatric disorders, PubMed and Scopus databases were searched using the following search terms: (vitamin C OR ascorbic acid) AND Alzheimer, (vitamin C OR ascorbic acid) AND Parkinson, (vitamin C OR ascorbic acid) AND Huntington, (vitamin C OR ascorbic acid) AND multiple sclerosis, (vitamin C OR ascorbic acid) AND amyotrophic sclerosis, (vitamin C OR ascorbic acid) AND depression, (vitamin C OR ascorbic acid) AND anxiety and (vitamin C OR ascorbic acid) AND schizophrenia, separately. The searching was limited to the last 10 years and human studies, but if none or a few human studies were found the criteria were expanded then to include in vitro or animal studies. 

The final search was conducted in April 2017. The titles and abstracts of the articles identified through the initial search were reviewed, and the irrelevant articles were excluded. The full texts of the remaining articles were reviewed to detect studies that did were not suitable for this review.

## 3. Vitamin C Transport Systems and Distribution in the Brain

Two basic barriers limit the entry of Vit C (being a hydrophilic molecule) into the central nervous system: the blood-brain barrier and the blood-cerebrospinal fluid barrier (CSF) [13]. Considering the whole body, ascorbic acid uptake is mainly conditioned by two sodium-dependent transporters from the SLC23 family, the sodium-dependent Vit C transporter type 1 (SVCT1) and type 2 (SVCT2). These possess similar structure and amino acid sequence, but have different tissue distribution. SVCT1 is found predominantly in apical brush-border membranes of intestinal and renal tubular cells, whereas SVCT2 occurs in most tissue cells [14,15]. SVCT2 is especially important for the transport of Vit C in the brain—it mediates the transport of ascorbate from plasma across choroid plexus to the cerebrospinal fluid and across the neuronal cell plasma membrane to neuronal cytosol [16]. Although dehydroascorbic acid (DHA) enters the central nervous system more rapidly than the ascorbate, the latter one readily penetrates CNS after oral administration. DHA is taken up by the omnipresent glucose transporters (GLUT), which have affinity to this form of Vit C [17,18]. GLUT1 and GLUT3 are mainly responsible for DHA uptake in the CNS [13]. Transport of DHA by GLUT transporter is bidirectional—each molecule of DHA formed inside the cells by oxidation of the ascorbate could be effluxed and lost. This phenomenon is prevented by efficient cellular mechanisms of DHA reduction and recycling in ascorbate [19]. Neurons can take up ascorbic acid using both described ways [20], whereas astrocytes acquire Vit C utilizing only GLUT transporters [21]. 

The brain has been found to belong to the organs of the highest ascorbate content, with neurons displaying the highest concentration of all the human organism and reaching 10 mmol/L [1,22]. Mefford et al. [23] and Milby et al. [24] showed high concentrations of Vit C in neuron-rich areas of hippocampus and neocortex in the human brain. Authors suggested that ascorbate content in above brain areas is as much as two-fold higher than in other regions. The difference in ascorbate content between neurons and glia appears to be significant [25]. It is postulated that in astrocytes and glial supported cells lacking the SVCT2, the uptake and reduction of DHA may be the only mechanism of ascorbate retention [26]. In addition to ascorbate motion in neurons and glial cells, it is also released from both types of cells. This release contributes to a certain extent to the homeostatic mechanism of extracellular ascorbate maintenance in the brain [15,19]. Moreover, the extracellular ascorbate concentration is regulated dynamically by glutamate release—increase in extracellular Vit C concentration causes heteroexchange with glutamate [27,28]. 

## 4. Vitamin C Function in Central Nervous System

It is well known that the main function of intracellular ascorbic acid in the brain is the antioxidant defense of the cells. However, vitamin C in the central nervous system (CNS) has also many non-antioxidant functions—it plays a role of an enzymatic co-factor participating in biosynthesis of such substances as collagen, carnitine, tyrosine and peptide hormones. It has also been indicated that myelin formation in Schwann cells could be stimulated by ascorbic acid [7,29].

The brain is an organ particularly exposed to oxidative stress and free radicals’ activity, which is associated with high levels of unsaturated fatty acids and high cell metabolism rate [16]. Ascorbic acid, being an antioxidant, acts directly by scavenging reactive oxygen and nitrogen species produced during normal cell metabolism [30,31]. In vivo studies demonstrated that the ascorbate had the ability to inactivate superoxide radicals—the major byproduct of fast metabolism of mitochondrial neurons [32]. Moreover, the ascorbate is a key factor in the recycling of other antioxidants, e.g., alpha-tocopherol (Vitamin E). Alpha-tocopherol, found in all biological membranes, is involved in preventing lipid peroxidation by removing peroxyl radicals. During this process α-tocopherol is oxidized to the α-tocopheroxyl radical, which can result in a very harmful effect. The ascorbate could reduce the tocopheroxyl radical back to tocopherol and then its oxidized form is recycled by enzymatic systems with using NADH or NADPH [33]. Regarding these facts, vitamin C is considered to be an important neuroprotective agent. 

One non-antioxidant function of vitamin C is its participation in CNS signal transduction through neurotransmitters [16]. Vit C is suggested to influence this process via modulating of binding of neurotransmitters to receptors as well as regulating their release [34,35,36,37]. In addition, ascorbic acid acts as a co-factor in the synthesis of neurotransmitters, particularly of catecholamines—dopamine and norepinephrine [26,38]. Seitz et al. [39] suggested that the modulating effect of the ascorbate could be divided into short- and long-term ones. The short-term effect refers to ascorbate role as a substrate for dopamine-β-hydroxylase. Vit C supplies electrons for this enzyme catalyzing the formation of norepinephrine from dopamine. Moreover, it may exert neuroprotective influence against ROS and quinones generated by dopamine metabolism [16]. On the other hand, the long-term effect could be connected with increased expression of the tyrosine hydroxylase gene, probably via a mechanism that entails the increase of intracellular cAMP [39]. It has been stated that the function of ascorbic acid as a neuromodulator of neural transmission may be also associated with amino acidic residues reduction [40] or scavenging of ROS generated in response to neurotransmitter receptor activation [34,41]. Moreover, some have studies showed that ascorbic acid modulates the activity of some receptors such as glutamate as well as γ-aminobutyric acid (GABA) ones [22,40,42,43,44]. Vit C has been shown to prevent excitotoxic damage caused by excessive extracellular glutamate leading to hyperpolarization of the *N*-methyl-d-aspartate (NMDA) receptor and therefore to neuronal damage [45]. Vit C inhibits the binding of glutamate to the NMDA receptor, thus demonstrating a direct effect in preventing excessive nerve stimulation exerted by the glutamate [26]. The effect of ascorbic acid on GABA receptors can be explained by a decrease in the energy barrier for GABA activation induced by this agent. Ascorbic acid could bind to or modify one or more sites capable of allosterically modulating single-channel properties. In addition, it is possible that ascorbic acid acts through supporting the conversion from the last GABA-bound closed state to the open state. Alternatively, ascorbic acid could induce the transition of channels towards additional open states in which the receptor adopts lower energy conformations with higher open probabilities [40,44].

There have also been reports concerning the effect of Vit C on cognitive processes such as learning, memory and locomotion, although the exact mechanism of this impact is still being investigated [26]. However, animal studies have shown a clear association between the ascorbate and the cholinergic and dopaminergic systems, they also suggested that the ascorbate can act as a dopamine receptor antagonist. This was also confirmed by Tolbert et al. [46], who showed that the ascorbate inhibits the binding of specific dopamine D1 and D2 receptor agonists.

Another non-antioxidant function of Vit C includes modulation of neuronal metabolism by changing the preference for lactate over glucose as an energy substrate to sustain synaptic activity. During ascorbic acid metabolic switch, this vitamin is released from glial cells and is taken up by neurons where it restraints glucose transport and its utilization. This allows lactate uptake and its usage as the primary energy source in neurons [47]. It was observed that intracellular ascorbic acid inhibited neuronal glucose usage via a mechanism involving GLUT3 [48].

Vit C is involved in collagen synthesis, which also occurs in the brain [26]. There is no doubt that collagen is needed for blood vessels and neural sheath formation. It is well recognized that vitamin C takes part in the final step of the formation of mature triple helix collagen. In this stage, ascorbic acid acts as an electron donor in the hydroxylation of procollagen propyl and lysyl residues [16]. The role of Vit C in collagen synthesis in the brain was confirmed by Sotiriou et al. [49]. According to these authors in mice deficient in SVCT2 ascorbate transporter, the concentration of ascorbate in the brain was below detection level. The animals died due to capillary hemorrhage in the penetrating vessels of the brain. Ascorbate-dependent collagen synthesis is also linked to the formation of the myelin sheath that surrounds many nerve fibers [26]. In vitro studies showed that ascorbate, added to a mixed culture of rat Schwann cells and dorsal root ganglion neurons, promoted myelin formation and differentiation of Schwann cells during formation of the basal lamina of the myelin sheath [7,29].

## 5. Role of Vitamin C in Neurodegenerative Diseases

Vit C is important for proper nervous system function and its abnormal concentration in nervous tissue is thought to be accompanied with neurological disorders. Studies have shown that disruption of vitamin C transport may cause brain damage in premature infants. Vit C was found to show alleviating effect on seizures severity as well as reducing influence on seizure-induced damage of hippocampus [8,9]. One of the recent studies also revealed that glutamate-induced negative changes in immature brain of rats were reduced by Vit C treatment [50]. Moreover, Vit C administration was shown to recover the colchicine-induced neuroinflammation-mediated neurodegeneration and memory impairments in rats [12] as well as ameliorate behavioral deficits and neuropathological alterations in rats exposed to aluminum chloride [11].

The fact that Vit C can neutralize superoxide radicals, which are generated in large amount during neurodegenerative processes, seems to support its role in neurodegeneration. Moreover, plasma and cellular Vit C levels decline steadily with age and neurodegenerative diseases are often associated with aging. An association of Vit C release with motor activity in central nervous system regions, glutamate-uptake-dependent release of Vit C, its possible role in modulation of *N*-methyl-d-aspartate receptor activity as well as ability to prevent peroxynitrite anion formation constitute further evidence pointing to the role of Vit C in neurodegenerative processes.

### 5.1. Alzheimer’s Disease

Alzheimer’s disease (AD) is the most common form of dementia, an incurable and progressive neurodegenerative disease, leading to far-reaching memory loss, cognitive decline and eventually death. There are two major forms of the AD disease: early onset (familial) and late onset (sporadic). Early-onset one is rare, accounting for less than 5% of all AD cases. Mutations in three genes, mainly amyloid precursor protein (21q21.3), presenilin-1 (14q24.3) and presenilin-2 (1q42.13), have been identified to be involved in the development of this form. Late-onset AD (LOAD) is common among individuals over 65 years of age. Although heritability of LOAD is high (79%), its etiology is considered to be polygenic and multifactorial. The apolipoprotein E ε4 allele (19q13.2) is the major known genetic risk factor for this form of AD. The E4/E4 genotype does not determine the occurrence of LOAD, but is a factor that increases susceptibility to this disease and lowers the age of disease onset. Moreover, a large number of genes have been suggested to be implicated in risk of late-onset Alzheimer’s, e.g., clusterin (8p21), complement receptor 1 (1q32), phosphatidylinositol binding clathrin assembly protein (11q14.2), myc box-dependent-interacting protein 1 (2q14.3), ATP binding cassette transporter 7 (19p13.3), membrane-spanning 4-domains, subfamily A (11q12.2), ephrin type-A receptor 1 (7q34), CD33 antigen (19q13.3), CD2 associated protein (6p12.3), sortilin-related receptor 1 (11q24.1), GRB2 associated-binding protein 2 (11q13.4–13.5), insulin-degrading enzyme (10q24), death-associated protein kinase 1 (DAPK1) or gene encoding ubiquilin-1 (UBQLN1) [51,52]. The list of genes associated with AD is still growing. For instance, in the recent study, Lee et al. revealed that single-nucleotide polymorphisms in six genes, including 3-hydroxybutyrate dehydrogenase, type 1 (*BDH1*), ST6 beta-galactosamide alpha-2,6-sialyltranferase 1 (*ST6GAL1*), RAB20, member RAS oncogene family (*RAB20*), PDS5 cohesin associated factor B *(PDS5B*), adenosine deaminase, RNA-specific, B2 (*ADARB2*), and SplA/ryanodine receptor domain and SOCS box containing 1 (*SPSB1*), were directly or indirectly related to conversion of mild cognitive impairment to AD [53]. 

A neuropathological lesions characteristic of AD include neurofibrillary tangles (composed of hyperphosphorylated and aggregated tau protein) accumulated in the neuronal cytosol as well as the extracellular plaque deposits of the β-amyloid peptide (Aβ), with their frequency correlating with declining cognitive measures [54]. Proteolytic cleavage of amyloid precursor polypeptide chain by secretases (mainly β- and γ-secretase) produces Aβ40 and Aβ42 peptides, which consist of 40 and 42 amino acids, respectively. The latter one, due to its hydrophobicity, is characterized by a greater tendency to form fibrils and is believed to be the main factor responsible for the formation of amyloid deposits [55]. However, Nagababu et al. suggested that the enhanced toxic effect observed for Aβ42 could be attributed to a greater toxicity of the 1–42 aggregates than the 1–40 ones of a comparable size distribution and not to the formation of larger fibrils [56]. According to Ott et al. [54] pre-aggregated Aβ42 peptide induces hyperphosphorylation and pathological structural changes of tau protein and thereby directly links the “amyloid hypothesis” to tau pathology observed in AD [54]. Although the pathogenesis of AD has not been fully understood yet, many studies have demonstrated that ROS and oxidative stress are implicated in disease progression. Aβ peptide was found to enhance the neuronal vulnerability to oxidative stress and cause an impairment of electron transport chain, whereas oxidative stress was shown to induce accumulation of Aβ peptide which subsequently promotes ROS production [16,22,57]. Bartzokis et al. in turn [58] suggested that myelin breakdown in vulnerable late-myelinating regions released oligodendrocyte- and myelin-associated iron that promoted the development of the toxic amyloid oligomers and plaques. There is also the “amyloid cascade-inflammatory hypothesis” which assumes that AD probably results from the inflammatory response induced by extracellular β-amyloid protein deposits, which subsequently become enhanced by aggregates of tau protein [59]. Moreover, recent research has suggested that AD might be a prion-like disease [60,61]. 

The role of Vit C in AD disease was studied in APP/PSEN1 mice carrying human AD mutations in the amyloid precursor protein (APP) and presenilin (PSEN1) genes (transgenic mouse model of Alzheimer’s disease) with partial ablation of vitamin C transport in the brain [9,62,63].

Warner et al. [9] demonstrated that decreased brain Vit C level in the 6-month-old SVCT2+/− APP/PSEN1 mice (obtained by crossing APP/PSEN1 bigenic mice with SVCT2+/− heterozygous knockout mice, which have the lower number of the sodium-dependent Vit C transporter) was associated with enhanced oxidative stress in brain, increased mortality, a shorter latency to seizure onset after kainic acid administration (10 mg/kg i.p.), and more ictal events following treatment with pentylenetetrazol (50 mg/kg i.p.). Furthermore, the authors reported that Vit C deficiency alone in SVCT2+/− mice increased the severity of kainic acid- and pentylenetetrazol-induced seizures [62]. According to another study even moderate intracellular Vit C deficiency displayed an important role in accelerating amyloid aggregation and brain oxidative stress formation, particularly during early stages of disease development. In 6-month-old SVCT2+/− APP/PSEN1 mice increased brain cortex oxidative stress (enhanced malondialdehyde, protein carbonyls, F2-isoprostanes) and decreased level of total glutathione as compared to wild-type controls were observed. Moreover, SVCT2+/− mice had elevated levels of both soluble and insoluble Aβ1-42 and a higher Aβ1-42/Aβ1-40 ratio. In 14-month old mice there were more amyloid-β plaque deposits in both hippocampus and cortex of SVCT2+/−APP/PSEN1+ mice as compared to APP/PSEN+ mice with normal brain Vit C level, whereas oxidative stress levels were similar between groups [62]. Ward et al. [63], in turn, showed that severe Vit C deficiency in Gulo−/− mice (lacking l-gulono-1,4-lactone oxidase (*Gulo*) responsible for the last step in Vit C synthesis) resulted in decreased blood glucose levels, oxidative damage to lipids and proteins in the cortex, and reduction in dopamine and serotonin metabolites in both the cortex and striatum. Moreover, Gulo−/− mice displayed a significant decrease in voluntary locomotor activity, reduced physical strength and elevated sucrose preference. All the above-mentioned behaviors were restored to control levels after treatment with Vit C (250 mg/kg, i.p.). The role of Vit C in preventing the brain against oxidative stress damage seems to be also proved by the recent study performed by Sarkar et al. [64]. The researchers share a view that cerebral ischemia-reperfusion-induced oxidative stress may initiate the pathogenic cascade leading eventually to neuronal loss, especially in hippocampus, with amyloid accumulation, tau protein pathology and irreversible Alzheimer’s dementia. Being the prime source of ROS generation, neuronal mitochondria are the most susceptible to damage caused by oxidative stress. The study proved it that l-ascorbic acid loaded polylactide nanocapsules exerted a protective effect on brain mitochondria against cerebral ischemia-reperfusion-induced oxidative injury [64]. Kennard and Harrison, in turn, evaluated the effects of a single intravenous dose of Vit C on spatial memory (using the modified Y-maze test) in APP/PSEN1 mice. The study was performed on APP/PSEN1 and wild-type (WT) mice of three age spans (3, 9 or 20 months). It was shown that APP/PSEN1 mice displayed no behavioral impairment as compared to WT controls, but memory impairment along with aging was observed in both groups. Vit C treatment (125 mg/kg, i.v.) improved performance in 9-month old APP/PSEN1 and WT mice, but improvements in short-term spatial memory did not result from changes in the neuropathological features of AD or monoamine signaling, as acute Vit C administration did not alter monoamine levels in the nucleus accumbens [65]. Cognitive-enhancing effects of acute intraperitoneal (i.p.) Vit C treatment in APP/PSEN1 mice (12- and 24-month-old) were investigated by Harrison et al. Vit C treatment (125 mg/kg i.p.) improved Y-maze alternation rates and swim accuracy in the water maze in both APP/PSEN1 and wild-type mice; but like in the previous study had no significant effect on the age-associated increase in Aβ deposits and oxidative stress, and did not also affect acetylcholinesterase (AChE) activity either, which was significantly reduced in APP/PSEN1 mice [66]. Murakami et al. [67] in turn reported that 6-month-treatment with Vit C resulted in reduced Aβ oligomer formation without affecting plaque formation, a significant decrease in brain oxidative damage and Aβ42/Aβ40 ratio as well as behavioral decline in an AD mouse model. Furthermore, this restored the declined synaptophysin and reduced the phosphorylation of tau protein at Ser396. 

Besides the presented roles, Vit C has also been suggested to prevent neurodegenerative changes and cognitive decline by protecting blood–brain barrier (BBB) integrity [68]. 

Kook et al., in the study performed on KO-Tg mice (generating by crossing 5 familial Alzheimer’s disease mutation (5XFAD) mice with mice lacking *Gulo*), found that oral Vit C supplementation (3.3 g/L of drinking water) reduced amyloid plaque burden in the cortex and hippocampus by ameliorating BBB disruption (via preventing tight junction structural changes) and morphological changes in the mitochondria [69]. This seems to be confirmed by other studies that proved that Vit C might affect levels of proteins responsible for the tightness of BBB, like tight junction-specific integral membrane proteins (occludin and claudin-5) as well as matrix metalloproteinase 9 (MMP-9). Allahtavakoli et al. demonstrated that in a rat stroke model Vit C administration (500 mg/kg; 5 h after stroke) significantly reduced BBB permeability by reducing serum levels of matrix metalloproteinase 9 [70]. Song et al. reported that Vit C (100 mg/kg i.p.) protected cerebral ischemia-induced BBB disruption by preserving the expression of claudin 5 [71], whereas Lin et al. observed that Vit C (500 mg/kg i.p.) prevented compression-induced BBB disruption and sensory deficit by upregulating the expression of both occludin and claudin-5 [72].

In the available literature, there were only few studies investigating the role of Vit C in AD disease in human and the existing ones have yielded equivocal results. 

Some studies have shown significantly lower plasma/serum Vit C level in AD patients as compared to healthy individuals, whereas others have found no difference [73,74]. However, meta-analysis performed by Lopes da Silva et al. proved significantly lower plasma levels of Vit C in AD patients [75]. It seems that the above discrepancies may result from the fact that not plasma but rather intracellular Vit C may be associated with AD. 

Generally, studies involving human participants are limited to assessing the effect of Vit C supplementation administrated with other antioxidants on AD course.

Arlt et al. [76] found that 1-month and 1-year co-supplementation of Vit C (1000 mg/day) with vitamin E (400 IU/day) increased their concentrations not only in plasma but also in cerebrospinal fluid (which reflects the Vit C status of the brain), while cerebrospinal fluid lipid oxidation was significantly reduced only after 1 year. However, vitamins’ supplementation did not have a significant effect on the course of AD [76]. These findings were aslo confirmed by the randomized clinical trial of Galasko et al. [77], which showed that treatment of AD patients for 16 weeks with vitamin E (800 IU/day) plus Vit C (500 mg/day) plus α-lipoic acid (900 mg/day) did not influence cerebrospinal fluid levels of Aβ42, tau and p181tau (widely accepted biomarkers related to amyloid or tau pathology), but decreased F2-isoprostane level (a validated biomarker of oxidative stress). Moreover, is should be emphasized that the above treatment increased risk of faster cognitive decline. This seems to be consistent with results of the recent study which revealed it that Vit C was a potent antioxidant within the AD brain, but it was not able to ameliorate other factors linked to AD pathogenesis as it was proved to be a poor metal chelator and did not inhibit Aβ42 fibrillation [78]. In the study considering an association between nutrient patterns and three brain AD-biomarkers, namely Aβ load, glucose metabolism and gray matter volumes (a marker of brain atrophy) in AD-vulnerable regions, it was found that the higher intake of carotenoids, vitamin A, vitamin C and dietary fibers was positively associated only with glucose metabolism [79]. 

On the other hand, a randomized control trial involving 276 elderly participants demonstrated that 16-week-co-supplementation of vitamin E and C with β-carotene significantly improved cognitive function (particularly with higher doses of β-carotene). Furthermore, the authors suggested that such a treatment markedly reduced plasma Aβ levels and elevated plasma estradiol levels [80]. Vit C and E co-supplementation for more than 3 years was also shown to be associated with a reduced prevalence and incidence of AD [81]. Moreover, an adequate Vit C plasma level seems to be associated with less progression in carotid intima-media thickness (C-IMT)—the greater C-IMT is suggested to be a risk factor in predicting cognitive decline in the general population, in the elderly population and in patients with Alzheimer’s disease. Polidori et al. showed significant decrease (with a linear slope) in Vit C level among old individuals with no or very mild cognitive impairment from the first to the fourth C-IMT quartile [82].

### 5.2. Parkinson’s Disease

Parkinson’s disease (PD) is a common long-term neurodegenerative movement disorder characterized by the progressive loss of substantia nigra dopaminergic neurons and consequent depletion of dopamine in the striatum. Dementia, depression and behavioral deficiencies are common symptoms in the advanced stages of the disease [22]. PD is pathologically heterogeneous, but abnormal aggregation of α-synuclein (α-syn) within neuronal perikarya (Lewy bodies) and neurites (Lewy neurites) are neuropathological (but not pathognomonic) hallmarks of this disease [83]. The primary cause of the neurodegenerative process underlying PD is still unknown. Only about 10% of PD cases have shown to be hereditary, whereas the rest are sporadic and result from complex interactions between environmental and common genetic risk factors. Monogenic PD with autosomal-dominant inheritance is caused by mutation in α-synuclein gene (*SNCA*) or leucine-rich repeat kinase 2 gene (*LRRK2*), whereas the form with autosomal recessive inheritance by mutations in the genes encoding Parkin 2 (*PARK2*), PTEN-induced putative kinase 1 (*PINK1*), protein deglycase DJ-1 (*PARK7*), and protein ATP13A2 (*PARK9*). However, many diverse genetic defects in other loci have been suggested to be associated with PD. Candidate genes which have been reported to be associated with PD include e.g., β-glucocerebrosidase (*GBA*), diacylglycerol kinase θ, 110kD (*GAK-DGKQ*), *SNCA*, human leukocyte antigen (*HLA*), *RAD51B, DYRK1A, CHCHD2, VPS35, RAB39B* or *TMEM230* [84,85]. Different mechanisms, including genomic factors, epigenetic changes, toxic factors, mitochondrial dysfunction, oxidative stress, neuroimmune/neuroinflammatory reactions, hypoxic-ischemic conditions, metabolic deficiencies and ubiquitin–proteasome system dysfunction, seem to be involved in PD pathogenesis [84,86,87,88,89,90,91,92]. Mitochondrial dysfunction has been shown to be linked to mutations in *PINK1* and *DJ1* genes [87,88]. Moreover, it is known that dopamine metabolism produces oxidant species, whereas oxidative stress participates in protein aggregation in PD [22,90,93]. Glutamate-mediated excitotoxicity has been proposed to be a further PD factor. It is also suggested that, like in the case of AD, PD might be a prion-like disease [94,95,96]. Olanow et al. [94] proposed the hypothesis that α-synuclein is a prion-like protein that can adopt a self-propagating conformation and thereby cause neurodegeneration. Scheffold et al. [97], in turn, reported that telomere shortening (one of the hallmarks of ageing) led to an acceleration of synucleinopathy and impaired microglia response and thereby might contribute to PD pathology. It is likely that not the above factors per se, but rather their synergistic interactions result in the development of the nigrostriatal damage in PD.

Vit C is believed to play a role in dopaminergic neuron differentiation. He et al. [98] in in vitro study found that Vit C enhanced the differentiation of midbrain derived neural stem cell towards dopaminergic neurons by increasing 5-hydroxymethylcytosine (5hmC) and decreasing histone H3 lysine 27 tri-methylation (H3K27m3) generation in dopamine phenotype gene promoters, which are catalyzed by ten-eleven-translocation 1 methylcytosine dioxygenase 1 (Tet1) and histone H3K27 demethylase (Jmjd3), respectively [98,99]. It seems that Vit C acts through regulation of Tet1 and Jmjd3 activities (it acts as a co-factor), since Tet1 and Jmjd3 knockdown/inhibition resulted in no effect of Vit C on either 5hmC or H3K27m3 in the progenitor cells [98]. In another in vitro study, it was shown that mouse embryonic fibroblasts cultured in Vit C-free medium displayed extremely low content of 5hmC, whereas treatment with Vit C resulted in a dose- and time-dependent increase in 5-hmC generation, which was not associated with any change in *Tet* genes expression. Additionally, it was found that treatment with another reducing agent as glutathione did not affect 5-hmC, whereas blocking Vit C entry into cells or knocking down *Tet* expression significantly reduced the effect of Vit C on 5-hmC [100].

Vit C is also believed to play an indirect role in α-syn oligomerization. Posttranslational α-syn modifications caused by oxidative stress, including modification by 4-hydroxy-2-nonenal, nitration and oxidation, have been implicated to promote oligomerization of α-syn, whereas Vit C as an antioxidant prevents this effect [22,101]. Jinsmaa et al. [102] found that treatment with Vit C attenuated Cu^2+^-mediated augmentation of 3,4-dihydroxyphenylacetaldehyde (DOPAL)-induced α-syn oligomerization in rat pheochromocytoma PC12 cells, but alone (without Cu^2+^) did not exert such an effect. Khan et al. showed, in turn, that Vit C supplementation (227.1 µM, 454.2 µM or 681.3 µM in diet, 21 days) caused a significant dose-dependent delay in the loss of climbing ability of PD Drosophila model expressing normal human α-syn in the neurons [103].

Moreover, Vit C is thought to be involved in neuroprotection against glutamate-mediated excitotoxicity occurring in PD. Ballaz et al. [104] in in vitro study performed on dopaminergic neurons of human origin showed that Vit C prevented cell death following prolonged exposure to glutamate. Glutamate induced toxicity in a dose-dependent way via the stimulation of α-amino-3-hydroxy-5-methyl-4-isoxazole propionic acid (AMPA) and metabotropic receptors and to a lesser degree by *N*-methyl-d-aspartate (NMDA) and kainate receptors, whereas Vit C (25–300 µM) administration protected cells against glutamate excitotoxity. The authors emphasized the fact that such a neuroprotection effect was dependent on the inhibition of oxidative stress, as Vit C prevented the pro-oxidant action of quercetin occurred over the course of prolonged exposure [104]. Vit C neuroprotection effect against dose-dependent glutamate-induced neurodegeneration in the postnatal brain was also confirmed by Shah et al. [50].

The effect of Vit C on dopamine system has also been observed. Izumi et al. [105] showed that PC12 cells treated with paraquat (50 µM, 24 h) displayed increased levels of cytosolic and vesicular dopamine, whereas pretreatment with Vit C (0.3–10 µM, 24 h) suppressed the elevations of intracellular dopamine and almost completely prevented paraquat toxicity.

Human studies have shown that Vit C deficiency among PD patients is widespread [106,107]. However, similarly like in the case of AD, not plasma but rather intracellular Vit C seems to be associated with PD. This could to be confirmed by the study performed by Ide et al. [108] who investigated the association between both lymphocyte and plasma Vit C levels in various stages of PD. Lymphocyte Vit C levels in patients with severe PD was significantly lower compared to those at less severe stages, whereas plasma Vit C levels showed a decreasing tendency; however that effect was not significant [108]. 

Although in the newest literature data, there are only a few human studies considering the role of Vit C treatment in PD, the existing ones give some evidences that Vit C treatment may have beneficial effect in PD course. A cohort study involving 1036 PD patients showed that dietary Vit C intake was significantly associated with reduced PD risk. However, it was not significant in a 4-year lagged analysis [109]. Quiroga et al., in turn, reported a case of a 66-year-old man with PD, pleural effusion and bipolar disorder who was found to have low serum Vit C and zinc levels. Intravenous replacement of both Vit C and zinc resulted in resolution of the movement disorder in less than 24 h [107]. The other case report concerned 83-year-old men with dementia, diabetes mellitus, hypertension, benign prostatic hypertension, paroxysmal atrial fibrillation, congestive heart failure and suspected PD. The man was treated with Vit C (200 mg) and zinc (4 mg), which resulted in complete resolution of periungual and gingival bleeding as well as palatal petechiae. Moreover, the man’s orientation and mental status were found to be markedly improved and no further delusions or agitations were observed [110]. 

Vit C was shown to increase l-dopa (3,4-dihydroxy-l-phenylalanine, one of the main drugs used in PD therapy) absorption in elderly PD patients. However, this effect was not observed in all patients but only in those with poor baseline *l*-dopa bioavailability [111]. Moreover, in vitro study performed by Mariam et al. revealed that Vit C is a strong inducer of *l*-dopa production from pre-grown mycelia of Aspergillus oryzae NRRL-1560 [112].

### 5.3. Huntington’s Disease

Huntington’s disease (HD) is a genetic, autosomal dominant disorder characterized by general neurodegeneration in brain with marked deterioration of medium-sized spiny neurons (MSNs) in the striatum [17,113]. HD is caused by a mutation (a CAG expansion) in the huntingtin gene (*HTT*), which results in an abnormal polyglutamine expansion in the huntingtin (HTT) protein and consequently HTT aggregation [113]. The mutant HTT alters intracellular Ca^2+^ homeostasis, induces mitochondrial dysfunction, disrupts intracellular trafficking and impairs gene transcription [114].

Clinically, HD is characterized by tripartite clinical features, namely progressive motor dysfunction (so-called choreic movements), neuropsychiatric symptoms and a variety of cognitive deficits [115,116]. Neuropathologically, HD is associated with a progressive, selective neuronal dysfunction and degeneration, especially in the both part of striatum (caudate and putamen) [117,118].

HD is known to be associated with a failure in energy metabolism, impaired mitochondrial ATP production and oxidative damage [113,119,120,121]. Other mechanisms, such as excitotoxicity, aberrant glutamatergic, dopaminergic and Ca^2+^ signaling mechanisms, metabolic damage, immune response, apoptosis as well as autophagy are also suggested to be involved in HD pathology [119,121,122,123,124]. 

Vit C flux from astrocytes to neurons during synaptic activity is regarded to be essential for protecting neurons against oxidative damage and modulation of neuronal metabolism, thus permitting optimal ATP production [119]. Under physiological conditions, Vit C is released from astrocytes to striatal extracellular fluid during increased synaptic activity. The enhancement of Vit C concentration in striatal extracellular fluid results in SVCT2 translocation to the plasma membrane and consequently Vit C uptake by neurons [119]. In neurons, Vit C is able to scavenge reactive oxygen species generated during synaptic activity and neuronal metabolism. As a result, Vit C is oxidized to dihydroascorbate, which is then released into the extracellular fluid and uptaken by neighboring astrocytes, where is subsequently turned back to a reduced form, which can be used again by neurons. Vit C can interact directly with reactive oxygen species but can also act as a co-factor in the reduction of other antioxidants as glutathione and α-tocopherol. Moreover, Vit C may function as a neuronal metabolic switch, which means that it is capable to inhibit glucose consumption and permit lactate uptake/use as a substrate to sustain synaptic activity. This function is not dependent on antioxidant activity of Vit C [47] and seems to be of great importance, taking into account that decreased expression of GLUT3 in both STHdhQ cells (striatal neurons derived from knock-in mice expressing mutant huntingtin; cell model of HD) and R6/2 mice (mouse model of HD) as well as impaired GLUT3 localization at the plasma membrane in HD cells were observed [125]. 

Unfortunately, the mechanism mentioned above does not work properly in HD. Abnormal Vit C flux from astrocytes to neurons was found both in R6/2 mice and STHdhQ cells. Acuña et al. proved that SVCT2 failed to reach the plasma membrane in cells expressing mutant Htt, which resulted in disturbed Vit C uptake by neurons [119]. Additionally, there is some evidence that altered glutamate transporter activity (GLT1—the protein primarily found on astrocytes and responsible for removing most extracellular glutamate), observed in HD, is related to deficient striatal Vit C release into extracellular fluid [126,127,128]. Miller et al. performed the study on R6/2 mice receiving ceftriaxone (200 mg/kg, once daily injection per 5 days)—a β-lactam antibiotic that selectively increases the expression of GLT1. To evaluate Vit C release in vivo voltammetry combined with corticostriatal afferent stimulation was used. R6/2 mice treated with saline displayed a marked decrease in striatal extracellular Vit C level compared to control group, whereas treatment with ceftriaxone restored striatal Vit C in R6/2 mice to control level and also improved the HD behavioral phenotype. It was also shown that intra-striatal infusion of GLT1 inhibitor (dihydrokainic acid or dl-*threo*-β-benzyloxyaspartate) blocked evoked striatal Vit C release [126]. Dorner et al., in turn, observed that cortical stimulation resulted in a rapid increase in Vit C release in both R6/2 and wild-type mice, but the response had a significantly shorter duration and smaller magnitude in R6/2 group. The researchers also measured striatal Vit C release in response to treatment with d-amphetamine (5 mg/kg)—a psychomotor stimulant known to release Vit C from corticostriatal terminals independently of dopamine. Both Vit C release and behavioral activation were diminished in R6/2 mice compared to wild-type ones. The authors concluded that the corticostriatal pathway was directly involved in behavior-related Vit C release and that this system was dysfunctional in HD [127]. It is thought that Vit C is released into striatal extracellular fluid as glutamate is uptaken—glutamate/Vit C heteroexchange. Consequently, Vit C level decreases while glutamate level increases in extracellular fluid of HD striatum owing to a downregulation of GLT1 [127,128]. Elevated glutamate level in synaptic gaps leads to abnormal signal transmission. 

In addition, it is also believed that long-term oxidative stress (one of the key players in HD progression) eliminates the ability of Vit C to modulate glucose utilization [125].

The effect of Vit C treatment on behavior-related neuronal activity was studied by Rebec et al. [129]. The authors showed that in the striatum of R6/2 mice impulse activity was consistently elevated compared to wild-type mice, whereas restoring extracellular Vit C to the wild-type level by Vit C treatment (300 mg/kg, 3 days) reversed this effect. This suggests Vit C involvement in normalization of neuronal function in HD striatum. In another study, the same researchers reported that regular injections of Vit C (300 mg/kg/day, 4 days/week) restored the behavior-related release of Vit C in striatum, which was associated with improved behavioral responding. Vit C treatment significantly attenuated the neurological motor signs of HD without altering overall motor activity [130]. 

Although studies performed on cell and animal models of HD appear to indicate the role of Vit C in HD course, to the best of our knowledge, in the newest literature there exists a lack of studies considering the role of Vit C or the effect of its supplementation in HD human subjects.

### 5.4. Multiple Sclerosis

Multiple sclerosis (MS) is a progressive demyelinating process considered as an autoimmune disease of unknown etiology. MS is characterized by infiltration of immune cells (in particular T cells and macrophages), demyelination (loss of myelin sheath that surrounds and protects nerve fibers allowing them to conduct electrical impulses) and axonal pathology resulting in multiple neurological deficits, which range from motor and sensory deficits to cognitive and psychological impairment [131,132]. The etiology of MS is still unknown, but it is suggested that genetic predisposition associated with environmental factors can lead to expression of the envelope protein of MS-associated retrovirus (MSRV) and thus trigger the disease [133]. Although pathogenesis of MS has not been fully clarified yet, either destruction by the immune system or a significant extent apoptosis, particularly apoptosis of oligodendroglia cells, are believed to be underlying mechanism. Oxidative/nitrosative stress and mitochondrial dysfunction are believed to contribute to the pathophysiology of MS [131,134,135,136,137]. 

Having regarded the presented facts, it seems to be justified that Vit C, being a very important brain antioxidant, may affect MS course. Vit C is known to affect numerous metabolic processes directly associated with immune system. Furthermore, Vit C-dependent collagen synthesis has also been linked to formation of the myelin sheath [7].

In the literature data, there are only a few studies considering association between MS and Vit C. However, the existing ones showed that MS patients displayed significantly lower Vit C level as compared to healthy individuals [135,136,138]. Besler et al. [138], in turn, observed an inverse correlation between the serum levels of Vit C and lipid peroxidation in MS patients. The authors concluded that decreased Vit C level, observed in MS patients during relapse of the disease, might be dependent on the elevated oxidative burden as reflected by increased lipid peroxidation. Hejazi et al. [139], in turn, found no significant difference between daily intake of Vit C (recorded from a 24-h dietary recall questionnaire for 3 days) in MS patients (*n* = 37) in comparison with healthy subjects. The intake of Vit C in both groups was below dietary reference intake (DRI), however in control group it was near the DRI value. 

An efficiency of antioxidant therapy in relapsing-remitting multiple sclerosis patients (*n* = 14) treated with complex of antioxidants and neuroprotectors with various mechanisms of action (oc-lipoic acid, nicotinamide, acetylcysteine, triovit beta-carotine, alpha-tocopheryl acetate, ascorbic acid, selenium, pentoxifylline, cerebrolysin, amantadine hydrochloride) during 1 month, 2 times a year was investigated by Odinak et al. [140]. The treatment resulted in significant reduction of relapse frequency, decrease of required corticosteroid courses and significantly reduced content of lipid peroxide products [140]. However, it should be underlined that Vit C was only one element of multicomponent treatment. However, in another study it was shown that intrahippocampal injection of Vit C (0.2, 1, 5 mg/kg, 7 days) improved memory acquisition of passive avoidance learning (PAL) in ethidium bromide-induced MS in rats. The injection of ethidium bromide caused significant deterioration of PAL, whereas treatment with Vit C at a dose of 5 mg/kg resulted in significant improvement in PAL [141].

Summing up, the possible role of Vit C in MS course remains to be explored.

### 5.5. Amyotrophic Lateral Sclerosis

Amyotrophic lateral sclerosis (ALS) is an incurable, chronic progressive neurodegenerative disease characterized by the degeneration of upper motor neurons in the motor cortex and lower motor neurons in the spinal cord and the brain stem [142]; the reason why only motor neurons are targeted remains unknown. ALS results in loss of power and function of skeletal muscles, which is reflected by difficulties in walking, using the arms, speaking and swallowing. ALS occurs in two forms: hereditary one, which is called familial (5–10% of ALS cases) and not hereditary one, called sporadic. Familial ALS is indistinguishable from the much more common sporadic form, but usually it begins at a slightly younger age. It is assumed that about 2% of all cases of ALS are caused by mutations in the gene encoding copper/zinc superoxide dismutase (SOD1) on chromosome 21, but the etiology of the remaining ALS cases is not fully understood. The course of ALS is variable, but usually relatively rapid. Most patients die, usually due to respiratory failure (respiratory muscles paralysis), within 3–5 years from the onset of symptoms [143].

Although the underlying causes of motor neuron degeneration remain still unknown, researchers have suggested a contribution of oxidative stress, mitochondrial dysfunction, glutamate-mediated excitotoxicity, cytoskeletal abnormalities, and protein aggregation [144]. Because of the above-presented facts and its activity-dependent release in the brain, it seems to be possible that Vit C may be involved in ALS pathogenesis. It appears to be confirmed by Blasco et al. who compared 1 H-NMR spectra of cerebrospinal fluid (CSF) samples collected from ALS patients (*n* = 44) and patients without a neurodegenerative disease. The authors found significantly higher Vit C level in the ALS group. Vit C, apart from being free radical scavenger, was suggested to modulate neuronal metabolism by reducing glucose consumption during episodes of glutamatergic synaptic activity and stimulating lactate uptake in neurons, which is consistent with lower lactate/pyruvate ratio seen in ALS patients [144].

However, in the available literature data, there are only a few studies evaluating an association between Vit C and ALS, and the existing ones have not proved its role in the course of this disease.

Nagano et al. [145] investigated the efficacy of Vit C treatment (0.8% *w*/*w* in the diet) in familiar ALS mice, administered before or after the onset of the disease. The mice treated with Vit C before disease onset survived significantly longer by 62% than the control. However, that treatment did not affect the mean age of onset appearance and administration after disease onset did not prolong survival. Netzahualcoyotzi and Tapia [146] found that the infusion of Vit C (20 mM), alone or in combination with glutathione ethylester, did not prevent the AMPA-induced motor alterations of the rear limbs and motor neuron degradation in rats. The pooled analysis of 5 large prospective studies of about 1100 ALS patients performed by Fitzgerald et al. showed that neither supplementation (even long-term) nor high dietary intake of Vit C affected risk of ALS [147]. Okamoto et al. [148] investigated the relationship between dietary intake of vegetables, fruit and antioxidants and the risk of ALS (153 ALS patients aged 18–81 years with disease duration of 3 years) in Japan. The study showed that a higher consumption of fruits and/or vegetables was associated with a significantly reduced risk of ALS. However, no significant dose-response relationship was observed between intake of beta-carotene, Vit C and vitamin E and the risk of ALS. Spasojević et al. [149], in turn, suggested that the use of Vit C could have an unfavorable effect in ALS patients. The researchers examined the effect of Vit C on the production of hydroxyl radicals in CSF obtained from sporadic ALS patients. Using electron paramagnetic resonance spectroscopy, the authors detected ascorbyl radicals in CSF of ALS patients, whereas in control CSF they were undetectable. Moreover, the addition of hydrogen peroxide to the CSF of ALS patients provoked further formation of ascorbyl as well as hydroxyl radicals ex vivo. Thus, it seems that herein Vit C may paradoxically induce pro-oxidative effects. This may result from the fact that Vit C is an excellent one-electron reducing agent that can reduce ferric (Fe^3+^) ion to ferrous (Fe^2+^) one, while being oxidized to ascorbate radical. In a Fenton reaction, Fe^2+^ reacts with H_2_O_2_ generating Fe^3+^ and a very strong oxidizing agent—hydroxyl radical. The presence of Vit C allows the recycling of Fe^3+^ back to Fe^2+^, which can subsequently catalyze the successive formation of hydroxyl radicals [1,150]. Moreover, it has also been shown that high concentrations of ascorbyl radical can reduce SOD activity. 

## 6. Role of Vitamin C in Psychiatric Disorders

Vit C is also believed to be involved in anxiety, stress, depression, fatigue and mood state in humans. It has been hypothesized that oral Vit C supplementation can elevate mood as well as reduce distress and anxiety. 

### 6.1. Depression

Depression (DP) is a mental disorder characterized by a number of basic symptoms like low mood, biological rhythm disorders, psychomotor slowdown, anxiety, somatic disorders as well other nonspecific symptoms [151]. It has a multifactorial etiology, with biological, psychological, social and lifestyle factors of important roles [152]. Several hypotheses have been proposed to explain the mechanisms underlying depression. Firstly, it is believed that depression is associated with disturbances of serotonin, norepinephrine and dopamine neurotransmission. Moreover, many observations have supported the involvement of GABAergic system in the pathomechanism of depression [153]. GABA level in plasma and CSF of patients suffering from depression was shown to be reduced [154,155] which points to its decreased synthesis in the brain. Recent data have suggested that chronic stress, via initiating changes in the hypothalamic-pituitary-adrenal axis and the immune system, acts as a trigger for the above-mentioned disturbance. For example, glucocorticoids and proinflammatory cytokines enhance the conversion of tryptophan to kynurenine thus leading to a decrease in the synthesis of brain serotonin (because less tryptophan is available for conversion to serotonin) and an increase in the formation of neurotoxic metabolites, e.g., glutamate antagonist quinolinic acid. The activity of the dopaminergic systems was also found to be reduced in response to inflammation [156]. Secondly, some genetic factors have been suggested to be implicated in depression etiology [157]. Thirdly, apoptosis of the brain cells seems to be involved in depression development, since a numerical and morphological alterations of astrocytes in patients with major depressive disorder were observed [158,159,160,161]. This may also be dependent, at least partially, on proinflammatory cytokine actions since quinolinic acid was shown to contribute to the increase in apoptosis of astrocytes or neurons [162,163].

Basing on several animal studies [153,155,164,165,166], there is preliminary evidence that Vit C exerts an antidepressant-like effect via:

1. modulation of monoaminergic systems [167] (e.g., Vit C was shown to activate the serotonin 1A (5-HT1A) receptor, this activation is a mechanism of action of many antidepressant, anxiolytic and antipsychotic drugs);
2. modulation of GABAergic systems (via activation of GABA_A_ receptors and a possible inhibition of GABA_B_ receptors) [155];
3. inhibition of *N*-methyl-d-aspartate (NMDA) receptors and l-arginine-nitric oxide (NO)-cyclic guanosine 3,5-monophosphate (cGMP) pathway—the blockade of NMDA receptor is associated with reduced levels of NO and cGMP, whereas reduction of NO levels within the hippocampus was shown to induce antidepressant-like effects [119];
4. blocking potassium (K^+^) channels—Vit C administration was shown to produce an antidepressant-like effect in the tail suspension test via K^+^ channel inhibition [119]; as K^+^ channels were reported to belong to the physiological targets of NO and cGMP in the brain, their inhibition plays a significant role in the treatment of depression;
5. activation of phosphatidylinositol-3-kinase (PI3K) and inhibition of glycogen synthase kinase 3 beta (GSK-3β) activity [112,119];
6. induction of heme oxygenase 1 expression—it is a candidate depression biomarker which may be a link factor between inflammation, oxidative stress and the biological as well functional changes in brain activity in depression; its decreased expression is associated with depressive symptoms [166,168];
7. since depression is well known to be associated with altered anti- and prooxidant profiles, Vit C may play antidepressant function also by its antioxidant properties [118,119].

The available literature data indicate that Vit C deficiency is very common in patients with depressive disorders. Gariballa [169] in a randomized, double blind, placebo-controlled trial observed that low Vit C status was associated with increased depression symptoms following acute illness in older people. Parameters were measured at baseline as well as after 6 weeks and 6 months. Patients with Vit C depletion had significantly increased symptoms of depression as compared to those with its higher concentrations both at baseline and at 6 weeks. Significantly lower serum Vit C level in patients with depression vs. healthy controls was also shown by Bajpai et al. [170] and Gautam et al. [171]. Moreover, in the latter study dietary supplementation of Vit C (1000 mg/day) along with vitamins A and E for a period of 6 weeks resulted in a significant reduction in depression scores [171]. Furthermore, a case-control study carried out on 60 male university students showed that subjects diagnosed with depression had significantly lower intake of Vit C than the healthy ones [172]. Similarly, in another case-control study involving 116 girls identified as having depressive symptoms, depression was negatively associated with Vit C intake, even after adjusting for confounding variables [173]. Rubio-López et al. [174], in turn, examined the relationship between nutritional intake and depressive symptoms in 710 Valencian schoolchildren aged 6–9 years and also observed that nutrient intake of Vit C was significantly lower in children with depressive symptoms. Additionally, prevalence of Vit C inadequacy (below dietary recommended intakes) was significantly higher in subjects with depressive symptoms. 

The efficacy of Vit C as an adjuvant agent in the treatment of pediatric major depressive disorder in a double-blind, placebo-controlled pilot trial was evaluated by Amr et al. [175]. Patients (*n* = 12) treated for six months with fluoxetine (10–20 mg/day) and Vit C (1000 mg/day) showed a significant decrease in depressive symptoms in comparison with the fluoxetine plus placebo group as measured by the Children’s Depression Rating Scale and Children’s Depression Inventory. No serious adverse effects were shown. Zhang et al. [176] in double-blind clinical trial investigated the effect of Vit C (500 mg twice daily) on mood in non-depressed acutely hospitalized patients. The applied therapy increased plasma and mononuclear leukocyte Vit C concentrations and was associated with a 34% reduction in mood disturbance (assessed with Profile of Mood States) [176]. Similarly, Wang et al. found that short-term Vit C (500 mg twice daily) treatment was associated with a 71% reduction in mood disturbance (assessed with Profile of Mood States) and a 51% reduction in psychological distress (assessed with Distress Thermometer) in acutely hospitalized patients with a high prevalence of hypovitaminosis C [177]. Khajehnasiri et al. [178] in a randomized, double-blind, placebo-controlled trial involving 136 depressed male shift workers observed, in turn, that Vit C administration (250 mg twice daily for 2 months) alone and in combination with omega-3 fatty acids significantly reduced the Beck Depression Inventory (BDI) score, however omega-3 fatty acid supplementation alone was more effective. Moreover, Vit C and omega-3 fatty acids supplementation alone (but not in combination) decreased significantly serum MDA levels. Fritz et al. [179] conducted a systematic review of human and observational studies assessing the efficiency of interventional Vit C as a contentious adjunctive cancer therapy and reported that it could improve quality of life, physical function, as well as prevent some side effects of chemotherapy, including fatigue, nausea, insomnia, constipation and depression. 

### 6.2. Anxiety

Anxiety is an adaptive response to uncertain threat, but it becomes pathological when is disproportionate to the threat, persists beyond the presence of the stressor, or is triggered by innocuous stimuli or situations. Similarly like in the case of depression, neurotransmitter system disruptions (namely GABA, serotonin and noradrenalin) as well as an impaired regulation of the hypothalamic-pituitary-adrenal axis are involved in anxiety disorders [180]. Furthermore, several studies have suggested a positive correlation between oxidative stress and anxiety-like behavior.

The growing evidence, which has been recently emerged, suggests that anxiety is associated with Vit C deficit, whereas Vit C supplementation could help reduce feeling of anxiety. The underlying mechanism is not fully understood yet, but Vit C seems to play this role by: regulating neurotransmitters’ activity, attenuating cortisol activity, preventing stress-induced oxidative damage and antioxidant defense in brain or some as yet undetermined effects on anxiety-related brain structures [181]. 

Kori et al. [182] observed that rats subjected to restrained stress (by placing in a wire mesh restrainer for 6 h per day for 21 days) displayed a significant increase in serum cortisol level with concomitant decrease in serum Vit C and E levels. Boufleur et al. [183], in turn, found decreased plasma Vit C levels in rats exposed to chronic mild stress. Interestingly, neonatal handling could prevent Vit C reduction in rats exposed to chronic mild stress in adulthood. Koizumi et al. [184] showed that Vit C status was critical for determining vulnerability to anxiety in a sex-specific manner. The study was performed on senescence marker protein–30/gluconolactones knockout mice (unable to synthesize Vit C) whose Vit C status was continuously shifted from adequate to depleted one (by providing a water with or without Vit C). It was observed that anxiety responses in the novelty-suppressed feeding paradigm were worse during Vit C depletion conditions, especially in females. Hughes et al. [181], in turn, reported that prolonged treatment with Vit C (approximately 80 mg/kg/day in drinking water, 83 days) markedly decreased anxiety-related behavior in the open field test in hooded rats. In another study, the same researchers examined the effect of Vit C treatment with three doses (61, 114 or 160 mg/kg/day in drinking water, 8 weeks) and observed that an anxiolytic effects of Vit C were displayed in higher frequencies of walking (with doses of 114 mg/kg/day and 160 mg/kg/day), higher frequencies of rearing (with dose of 61 mg/kg/day) and lower frequencies of grooming (with dose of 61 mg/kg/day) in the open-field as well as more frequent occupation of the open arms in the elevated plus-maze (with dose of 61 mg/kg/day). The authors concluded that anxiolytic effects of Vit C were more typical of the lowest dose and it was to some extent dependent on anxiety intensity [185]. The effect of Vit C on adrenal gland function (an element of the stress response system) was investigated by Choi et al. [186]. An adrenalectomized (ADX) and non-ADX rats were treated with Vit C (25 or 100 m/kg, 7 days) and subsequently subjected to both Vit C treatment and electroshock stress for next 5 days. Vit C supplementation reduced corticosterone level in non-ADX rats. Stress decreased the mean value of rearing frequency in both non-ADX and ADX rats, whereas Vit C partially attenuated this effect in non-ADX group. Moreover, Vit C treatment decreased adrenocorticotropic hormone in both groups and significantly reduced freezing time increased by stress. The authors suggested that the alleviating effect of Vit C on stress-related rearing behavior was exerted via modulation of corticosterone, whereas the effect on freezing behavior via modulation of corticotropin-releasing hormone or adrenocorticotropin-releasing hormone [186]. Puty et al. [187] in turn suggested that Vit C plays anxiolytic-like effect via affecting serotonergic system. The researchers evaluated the protective effect of Vit C against methylmercury (MeHg)-induced anxiogenic-like effect in zebrafish. MeHg produced a marked anxiogenic effects in the light/dark box test, which was accompanied by a decrease in the extracellular levels of serotonin as well an increase in its oxidized metabolite tryptamine-4,5-dione, whereas pretreatment with Vit C (2 mg/g, i.p.) prevented such alterations. Furthermore, Angrini and Leslie [188] found that pretreatment with Vit C (100 mg or 200 mg/kg) could attenuate, especially the higher dose, behavioral and anxiogenic effects of prolonged exposure to noise (100 dB for 2 months, 5 days/week, 4 h daily) on male laboratory mice. 

Although there are only a few studies considering the effects of vitamin C on anxiety and stress responses in humans, the existing ones seem to provide promising results. 

De Oliveira et al. [189] examined the effects of short-term oral Vit C supplementation (500 mg/day, 14 days) in high school students (*n* = 42) in a randomized, double-blind, placebo-controlled trial. The treatment led to higher plasma Vit C concentration that was associated with reduced anxiety levels evaluated with BIA (Beck Anxiety Inventory). Moreover, the Vit C supplementation had positive effect on the heart rate. Gautam et al. [171] observed that patients with generalized anxiety disorder had significantly lower Vit C levels in comparison with healthy controls, whereas 6-week vitamins supplementation (vitamin C accompanied with A and E) led to a significant reduction in anxiety scores [171]. Mazloom et al. [190], in turn, showed that short-term supplementation of Vit C (1000 mg/day) reduced anxiety levels (evaluated basing on Depression Anxiety Stress Scales 21-item) in diabetic patients. This effect was exerted through alleviating oxidative damage. Furthermore, recently performed a systematic review also showed that high-dose Vit C supplementation was effective in reducing anxiety as well as stress-induced blood pressure increase [191].

### 6.3. Schizophrenia

Schizophrenia is a severe and complex neuropsychiatric disorder that affects 1% of the population worldwide [192,193,194]. Symptoms of schizophrenia are described as “positive” (also so-called productive) and “negative” ones: the first include hallucinations, paranoia and delusions, while negative examples are: limited motivation, impaired speech, weakening and social withdrawal. These symptoms usually appear in early adulthood and often persist in about three-fourths of patients despite optimum treatment [192]. Some authors have suggested that insufficient dopamine level due to the loss of dopamine producing cells may lead to schizophrenia [195]. On the other hand, it has been postulated that schizophrenia has been linked to hyperactivity of brain dopaminergic systems that may reflect an underlying dysfunction of NMDA receptor-mediated neurotransmission [194]. Furthermore, there is the increasing evidence that several physiological mechanisms such as oxidative stress, altered one carbon metabolism and atypical immune-mediated responses may be involved in schizophrenia pathomechanism [192,196].

Hoffer [197] summarized in the review study the evidence showing that among others Vit C deficiency could worsen the symptoms of schizophrenia and that large doses of this vitamin could improve the core metabolic abnormalities predisposing some people to development of this disease. According to the author, it is probable that the pathologic process responsible for schizophrenia could increase ascorbic acid utilization. Sarandol et al. [198] also noted lower levels of serum Vit C as compared to control group, but this was not regarded as a statistically significant difference. Moreover, a 6-week-long antipsychotic treatment did not modify the concentration of this vitamin. The authors explained that other factors, such as nutrition, physical activity, etc., might be the reason for the discrepancy between the results of their research and other studies. Similarly, Young et al. [199] observed only a slight decrease in Vit C levels in schizophrenic group vs. control one; but interestingly, a highly significant increase in Vit C level in the control female group as compared to both control as well as schizophrenic male group was observed. The authors pointed out that this information might be relevant particularly in the light of recent reports that the risk of schizophrenia is higher in men than women. The reduced supply of Vit C with the diet in patients with schizophrenia was noted by Konarzewska et al. [200].

The review of Magalhães et al. revealed that the implementation of Vit C as a low-molecular-weight antioxidant alleviated the effects of free radicals in the treatment of schizophrenia [201]. According to Bentsen et al. [202] membrane lipid metabolism and redox regulation may be disturbed in schizophrenia. These authors conducted a study aiming at examination of the clinical effect of adding vitamins E + C to antipsychotics (D_2_ receptor antagonists). Patients with schizophrenia or related psychoses received Vit C (1000 mg/day) along with vitamin E (364 mg/day) for 16 weeks. Vitamins impaired the course of psychotic symptoms, especially of persecutory delusions. The authors pointed to the usefulness of supplementation of antioxidant vitamins as agents alleviating some side effects of antipsychotic drugs. This was also confirmed by the next study involving schizophrenia patients treated with haloperidol [203]. Classical antipsychotics like haloperidol are suggested to increase oxidative stress and oxidative cell injury in brain, which may influence the course as well as treatment effects of schizophrenia. In this study, chronic haloperidol treatment connected with supplementation of a combination of ω-3 fatty acids and vitamins E and C showed a significant beneficial effect on schizophrenia treatment as measured by SANS (Simpson Angus Scale) and BPRS (Brief Psychiatric Rating Scale) scales. BPRS total score and subscale scores as well as SANS scores were significantly improved starting from the 4th week of treatment. Moreover, in patients with schizophrenia after 16 weeks of treatment, serum Vit C levels were almost twice as high as at the beginning of the study. These results supported the hypothesis of a beneficial effect of the applied supplementation both on positive and negative symptoms of schizophrenia as well as the severity of side effects induced by haloperidol [203]. Heiser et al. [204] also stated that reactive oxygen species (ROS) were involved in the pathophysiology of psychiatric disorders such as schizophrenia. Their research demonstrated that antipsychotics induced ROS formation in the whole blood of rats, which could be reduced by the application of vitamin C. The aim of their study was to demonstrate the effects of clozapine, olanzapine and haloperidol at different doses (18, 90 and 180 μg/mL) on the formation of ROS in the whole blood by using electron spin resonance spectroscopy. To demonstrate the protective capacity of Vit C the blood samples were incubated the highest concentration of each drug with Vit C (1 mM) for 30 min. Olanzapine caused significantly greater ROS formation vs. control under all treatment conditions, while in the case of haloperidol and clozapine only two higher concentrations resulted in significantly increased ROS formation. Vitamin C reduced the ROS production of all tested drugs, but for olanzapine the attenuating effect did not reach a significant level.

A relatively novel approach as for the role of Vit C in etiology and treatment of schizophrenia was presented by Sershen et al. [193]. According to the researchers, deficits in *N*-methyl-d-aspartate receptor (NMDAR) function are linked to persistent negative symptoms and cognitive deficits in schizophrenia. This hypothesis is supported by the fact that the flavoprotein *D*-amino acid oxidase (DAO) was shown to degrade the gliotransmitter *D*-Ser, a potent activator of *N*-methyl-d-aspartate-type glutamate receptors, while a lot of evidence has suggested that DAO, together with its activator, G72 protein, may play a key role in the pathophysiology of schizophrenia. Furthermore, in a postmortem study the activity of DAO was found to be two-fold higher in schizophrenia subjects [205]. Sershen et al. [193] showed that acute ascorbic acid dose (300 mg/kg i.p.) inhibited PCP-induced and amphetamine-induced locomotor activity in mouse model, which was further attenuated in the presence of D-serine (600 mg/kg). The authors suggested that this effect could result from the Vit C-depended changes in dopamine carrier-membrane translocation and/or altered redox mechanisms that modulate NMDARs. However, this issue needs to be further investigated.

## 7. Conclusions

**Figure 2:**
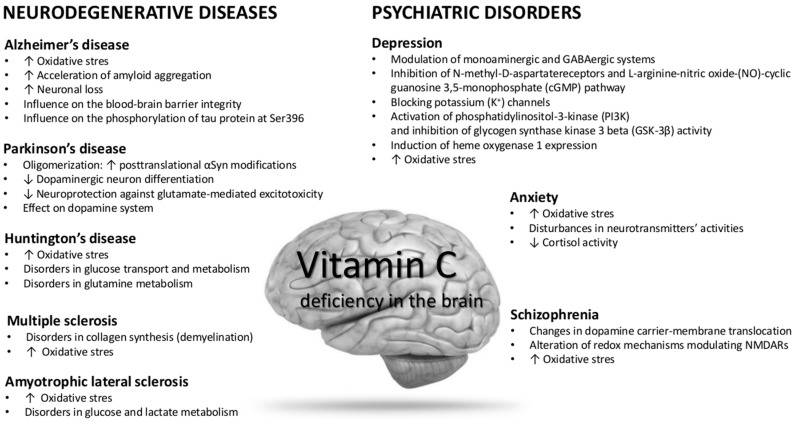
The main potential consequences of brain Vit C deficiency in the course and pathogenesis of neurological disorders.

The crucial role of Vit C in neuronal maturation and functions, neurotransmitter action as well as responses to oxidative stress is well supported by the evidences presented in this review (Figure 2).

The aforementioned animal studies confirmed the usefulness of using of Vit C in the treatment of neurological diseases, both neurodegenerative and psychiatric ones. Only in the case of ALS, the possible unfavorable effects were suggested. However, studies on the role of Vit C in the course of neurological disorders in human are limited and the existing ones have aimed mostly at evaluating the effect of Vit C supplementation (often co-supplementation with other agents). Recently, a tendency toward using administration of large doses of Vit C as an adjuvant in curing of many diseases was observed. Unfortunately, in the available literature there is a lack of studies considering this issue in the context of neurological disorders. 

In conclusion, the future studies concerning the question if Vit C could be a promising adjuvant in therapy of neurodegenerative and/or psychiatric disorders in humans, seem to be advisable.
